# Supplementary material for: Biochemical and Functional Characterization of the Three Zebrafish Transglutaminases 2
Source: Int J Mol Sci. 2023 Jul 27;24(15):12041. doi: 10.3390/ijms241512041 (PMC10419279; doi:10.3390/ijms241512041)
Supplement: Supplementary file 1 [file ijms-24-12041-s001.zip › ijms-2517881-supplementary.pdf]

		1	2	3	4	5	6	7
cloned	1		100.00	99.41	98.97	98.68	98.68	98.24
SAT	2	100.00		99.41	98.97	98.68	98.68	98.24
Tuebingen(reference)	3	99.41	99.41		98.97	98.97	98.68	98.24
CG1	4	98.97	98.97	98.97		98.53	98.24	97.50
AB	5	98.68	98.68	98.97	98.53		99.71	98.97
CG2	6	98.68	98.68	98.68	98.24	99.71		99.26
T5D	7	98.24	98.24	98.24	97.50	98.97	99.26	

Supplementary Table S1. Nucleotide similarity matrix of zTG2a. The nucleotide zTG2a sequence obtained with DNA sequencing from zebrafish embryos cDNA was compared with the zTG2a sequences of other publicly available zebrafish strains genome. The AB2, CB and NA strains were excluded from the analysis, as the complete CDS of zTG2a was not available.

		1	2	3	4	5	6	7
cloned	1		100.00	99.66	99.51	98.87	98.58	98.43
SAT	2	100.00		99.66	99.51	98.87	98.58	98.43
Tuebingen(reference)	3	99.66	99.66		99.31	98.92	98.53	98.28
CG1	4	99.51	99.51	99.31		98.82	98.48	98.33
AB	5	98.87	98.87	98.92	98.82		99.61	99.26
CG2	6	98.58	98.58	98.53	98.48	99.61		99.46
T5D	7	98.43	98.43	98.28	98.33	99.26	99.46	

Supplementary Table S2. Aminoacidic similarity matrix of zTG2a. The nucleotide zTG2a sequence was obtained with DNA sequencing from zebrafish embryos cDNA and the derived aminoacidic sequence was compared with the zTG2a sequences of other publicly available zebrafish strains genome. The AB2, CB and NA strains were excluded from the analysis, as the complete CDS of zTG2a was not available.

		1	2	3	4	5	6	7	8	9
NA	1		99.31	99.85	98.82	99.02	99.07	99.07	99.07	98.92
AB	2	99.31		99.36	99.22	99.61	99.66	99.66	99.66	99.36
CG2	3	99.85	99.36		98.87	99.07	99.12	99.12	99.12	98.97
T5D	4	98.82	99.22	98.87		99.22	99.26	99.26	99.26	98.68
cloned	5	99.02	99.61	99.07	99.22		99.95	99.95	99.95	99.17
SAT	6	99.07	99.66	99.12	99.26	99.95		100.00	100.00	99.22
AB2	7	99.07	99.66	99.12	99.26	99.95	100.00		100.00	99.22
Tuebingen(reference)	8	99.07	99.66	99.12	99.26	99.95	100.00	100.00		99.22
CB	9	98.92	99.36	98.97	98.68	99.17	99.22	99.22	99.22	

Supplementary Table S3. Nucleotide similarity matrix of zTG2b. The nucleotide zTG2b sequence obtained with DNA sequencing from zebrafish embryos cDNA was compared with the zTG2b sequences of other publicly available zebrafish strains genome.

		1	2	3	4	5	6	7	8	9
SAT	1		100.00	100.00	99.56	99.41	99.71	99.85	99.41	99.41
AB2	2	100.00		100.00	99.56	99.41	99.71	99.85	99.41	99.41
Tuebingen(reference)	3	100.00	100.00		99.56	99.41	99.71	99.85	99.41	99.41
NA	4	99.56	99.56	99.56		99.85	99.85	99.41	99.56	99.26
CG2	5	99.41	99.41	99.41	99.85		99.71	99.26	99.41	99.12
AB	6	99.71	99.71	99.71	99.85	99.71		99.56	99.71	99.41
cloned	7	99.85	99.85	99.85	99.41	99.26	99.56		99.26	99.26
CB	8	99.41	99.41	99.41	99.56	99.41	99.71	99.26		99.12
T5D	9	99.41	99.41	99.41	99.26	99.12	99.41	99.26	99.12	

Supplementary Table S4. Aminoacidic similarity matrix of zTG2b. The nucleotide zTG2b sequence was obtained with DNA sequencing from zebrafish embryos cDNA and the derived aminoacidic sequence was compared with the zTG2b sequences of other publicly available zebrafish strains genome.

		1	2	3	4	5	6	7	8
Tuebingen(reference)	1		99.71	99.71	99.71	99.71	100.00	99.57	99.57
SAT	2	99.71		100.00	100.00	100.00	99.71	99.57	99.28
CG1	3	99.71	100.00		100.00	100.00	99.71	99.57	99.28
AB2	4	99.71	100.00	100.00		100.00	99.71	99.57	99.28
AB	5	99.71	100.00	100.00	100.00		99.71	99.57	99.28
cloned	6	100.00	99.71	99.71	99.71	99.71		99.57	99.57
CG2	7	99.57	99.57	99.57	99.57	99.57	99.57		99.71
T5D	8	99.57	99.28	99.28	99.28	99.28	99.57	99.71	

Supplementary Table S5. Nucleotide similarity matrix of zTG2like. The nucleotide zTG2like sequence obtained with DNA sequencing from zebrafish embryos cDNA was compared with the zTG2like sequences of other publicly available zebrafish strains genome.

		1	2	3	4	5	6	7	8
SAT	1		100.00	100.00	99.28	99.47	99.47	99.18	99.86
CG1	2	100.00		100.00	99.28	99.47	99.47	99.18	99.86
AB2	3	100.00	100.00		99.28	99.47	99.47	99.18	99.86
CG2	4	99.28	99.28	99.28		99.52	99.52	99.52	99.42
Tuebingen(reference)	5	99.47	99.47	99.47	99.52		100.00	99.33	99.62
cloned	6	99.47	99.47	99.47	99.52	100.00		99.33	99.62
T5D	7	99.18	99.18	99.18	99.52	99.33	99.33		99.23
AB	8	99.86	99.86	99.86	99.42	99.62	99.62	99.23	

Supplementary Table S6. Aminoacidic similarity matrix of zTG2like. The nucleotide zTG2like sequence was obtained with DNA sequencing from zebrafish embryos cDNA and the derived aminoacidic sequence was compared with the zTG2like sequences of other publicly available zebrafish strains genome.

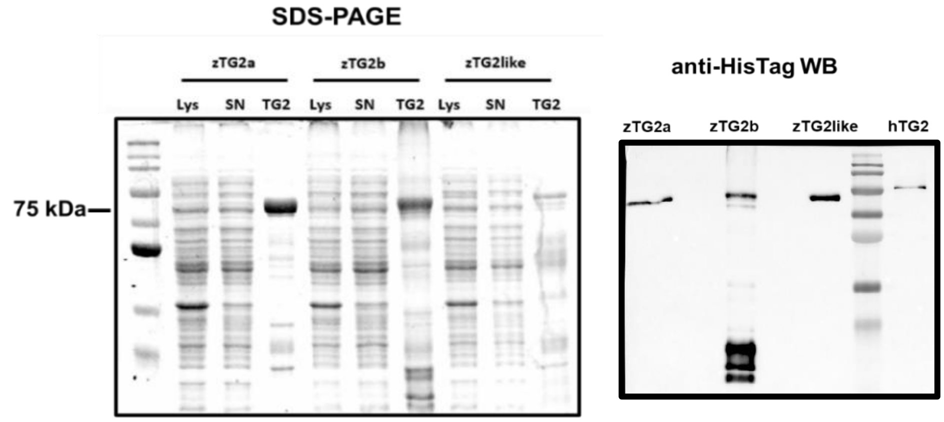

Supplementary Figure S1.

Recombinant zTGs2 protein production and purification. The 3 affinity-purified zTGs2 were analysed in SDS-PAGE (left panel) and Western blot (right panel). The total lysate (Lys) and soluble protein fraction (SN) are also shown in the protein gel. The three zTGs2 recombinant proteins were detected in Western blot with an anti-6HisTag antibody, together with the recombinant hTG2 as positive control.

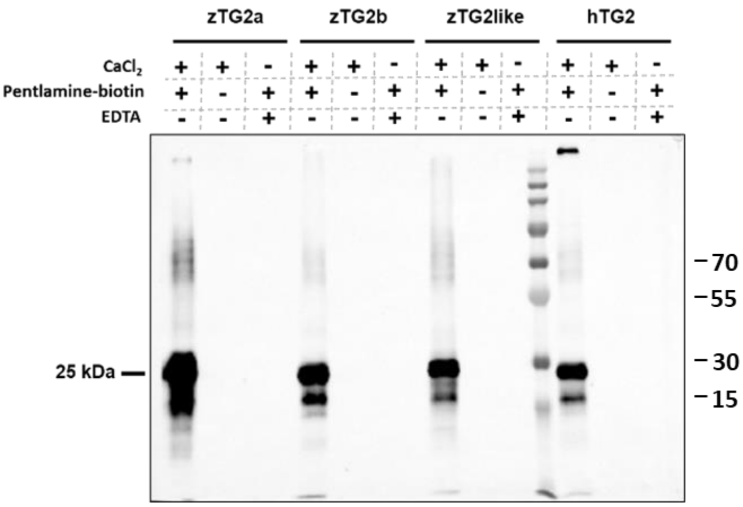

Supplementary Figure S2.

Transamidation activity in assay. The zTG2-catalysed incorporation of pentylamine biotin into DMC was detected by HRP-conjugated streptavidin in Western Blot. Human TG2 was also tested as a positive control.

HS1

**hTG2**	590	KIRILGEPKQKRK	602
**zTG2a**	583	IVNILGVPKVGRN	595
**zTG2b**	582	KIRILGEPKENRK	594
**zTG2like**	586	HVKIIGNAIVSRK	598

(a)

HS2

**hTG2**	202	NPKFLKNAGRDCSRRSSPVYVGR	222
**zTG2a**	196	SPNYISDAALDCSERKNAVYVTR	216
**zTG2b**	192	NPKHRRNPAKDCSGRRNVIYVTR	212
**zTG2like**	198	SPAALKNSEMDIFNRASPVYVSR	218

(b)

**hTG2**	261	LRRWKN	266
**zTG2a**	255	LRQWSN	260
**zTG2b**	252	LRTWDR	257
**zTG2like**	258	LRRWSE	263

(c)

Supplementary Figure S3.

TG2 Heparan-binding sites protein homology. Multiple protein sequence alignment performed with MUSCLE of three putative heparan-binding sites. a) Protein sequence alignment of hTG2 and zTGs2 of the HS1 described by Wang et co-workers (2012), comprising the KQKRQ peptide proposed by Lortat-Jacob and co-workers (2012). b) Protein sequence alignment of hTG2 and zTGs2 of the HS2 described by Wang et co-workers as the true region responsible for the interaction. c) Protein sequence alignment of hTG2 and zTGs2 of the peptide identified as the best candidate HBS according to the publications of Teesalu and co-workers (2012) and Lortat-Jacob and co-workers. The basic amino acids described as the key residues for the binding are highlighted in green. The conserved residues are in bold and highlighted in red.

Supplementary materials and methods

SDS-PAGE and Western blot of TGs2 recombinant proteins

10 nanograms of each recombinant affinity-purified protein were tested in western blot (WB) using an anti-hexahistidine Tag (6His-Tag) antibody. The samples were denatured for 5 minutes with loading buffer in boiling water and resolved on SDS-polyacrylamide gel. The proteins were transferred with Trans-Blot SD Semi-Dry Transfer Cell (Bio-Rad) onto a nitrocellulose membrane. After a blocking step of 45 minutes with 5% skim milk in PBS at room temperature (RT), the membrane was incubated with the for 1 hour with HRP-conjugated anti-6His-Tag antibody diluted in the blocking buffer in volumetric ratio 1:5000. After three washes with PBS-Tween 20 0.1% and three with PBS, the membrane was developed with Pierce™ ECL Western Blotting Substrate (Thermo Scientific, cat#32209) and imaged with ChemiDoc™ MP Imaging System (Bio-Rad).

In solution zTGs2 transamidation activity assay (supplementary Information)

In solution transamidation activity assay was performed by testing the incorporation of pentylamine-biotin catalysed by 100 ng of affinity-purified TG2 into 3 µg of DMC substrate in three conditions: 50 µL of reaction mix (100 mM Tris-HCl, pH 8.0, 0.2 mM EZ-Link™ Pentylamine-Biotin, 10 mM DTT, 5 mM CaCl2); 50 µL of reaction mix without 0.2 mM pentylamine-biotin; and 50 µL of reaction mix with 5 mM EDTA instead of CaCl2, as a negative control. The reaction was allowed to proceed for 1 hour at 37°C. Then, the reaction mixture was denatured by heating for 5 minutes in boiling water in Laemmli buffer and resolved on SDS-polyacrylamide gel. The proteins were transferred with Trans-Blot SD Semi-Dry Transfer Cell onto a nitrocellulose membrane. After a blocking step of 45 minutes with 5% skim milk in PBS at RT, three washes with PBS-Tween 0.1% and three with PBS, the membrane was incubated for 1 hour at RT with HRP-conjugated streptavidin diluted in PBS-BSA 1% in volumetric ratio 1:200. After three washes with PBS-Tween 20 0.1% and three with PBS, the membrane was developed with Pierce™ ECL Western Blotting Substrate and imaged with ChemiDoc™ MP Imaging System.
